# Mathematical modelling for antibiotic resistance control policy: do we know enough?

**DOI:** 10.1186/s12879-019-4630-y

**Published:** 2019-11-29

**Authors:** Gwenan M. Knight, Nicholas G. Davies, Caroline Colijn, Francesc Coll, Tjibbe Donker, Danna R. Gifford, Rebecca E. Glover, Mark Jit, Elizabeth Klemm, Sonja Lehtinen, Jodi A. Lindsay, Marc Lipsitch, Martin J. Llewelyn, Ana L. P. Mateus, Julie V. Robotham, Mike Sharland, Dov Stekel, Laith Yakob, Katherine E. Atkins

**Affiliations:** 1 Department of Infectious Disease Epidemiology, Faculty of Epidemiology and Population Health, London School of Hygiene and Tropical Medicine (LSHTM), London, UK; 2 Department of Mathematics, Simon Fraser University, Burnaby, Canada; 3 Department of Infection Biology, Faculty of Infectious and Tropical Diseases, LSHTM, London, UK; 4 Nuffield Department of Medicine, University of Oxford, Oxford, UK; 5 Faculty of Biology, Medicine and Health, The University of Manchester, Manchester, UK; 6 Department of Health Services Research and Policy, Faculty of Public Health and Policy, LSHTM, London, UK; 7 Wellcome Trust, London, UK; 8 Big Data Institute, Li Ka Shing Centre for Health Information and Discovery, Nuffield Department of Medicine, University of Oxford, Oxford, UK; 9 Institute for Infection and Immunity, St George’s, University of London, Cranmer Terrace, London, UK; 10 Center for Communicable Disease Dynamics, Department of Epidemiology, Harvard T. H. Chan School of Public Health, Boston, MA USA; 11 Department of Global Health and Infection, Brighton and Sussex Medical School, Brighton, UK; 12 Population Sciences and Pathobiology Department, Royal Veterinary College, London, UK; 13 Modelling and Economics Unit, National Infection Service, Public Health England, London, UK; 14 Paediatric Infectious Disease Research Group, St George’s University of London, London, UK; 15 School of Biosciences, University of Nottingham, Loughborough, UK; 16 Department of Disease Control, Faculty of Infectious and Tropical Diseases, LSHTM, London, UK; 17 Centre for Global Health Research, Usher Institute for Population Health Sciences and Informatics, The University of Edinburgh, Edinburgh, UK

**Keywords:** Dynamic modelling, Antibiotic resistance (ABR), Antimicrobial resistance (AMR), Decision-making

## Abstract

**Background:**

Antibiotics remain the cornerstone of modern medicine. Yet there exists an inherent dilemma in their use: we are able to prevent harm by administering antibiotic treatment as necessary to both humans and animals, but we must be mindful of limiting the spread of resistance and safeguarding the efficacy of antibiotics for current and future generations. Policies that strike the right balance must be informed by a transparent rationale that relies on a robust evidence base.

**Main text:**

One way to generate the evidence base needed to inform policies for managing antibiotic resistance is by using mathematical models. These models can distil the key drivers of the dynamics of resistance transmission from complex infection and evolutionary processes, as well as predict likely responses to policy change in silico. Here, we ask whether we know enough about antibiotic resistance for mathematical modelling to robustly and effectively inform policy. We consider in turn the challenges associated with capturing antibiotic resistance evolution using mathematical models, and with translating mathematical modelling evidence into policy.

**Conclusions:**

We suggest that in spite of promising advances, we lack a complete understanding of key principles. From this we advocate for priority areas of future empirical and theoretical research.

## Background

Mathematical modelling is a tool that allows us to integrate our mechanistic understanding of biological processes—such as the spread of antibiotic resistance (ABR)—in a precise and logical structure. A correctly-specified model can not only reproduce the empirical patterns that we observe, but also enable us to predict how changing conditions may impact upon real-world outcomes. Since ABR is a priority issue for global health, policymakers are increasingly concerned about how best to manage the spread of ABR, and are engaged in designing new guidelines and policies for doing so. Mathematical modelling has the potential to help inform these policies because it can quickly and inexpensively predict the outcomes of different actions, including inaction. Here we discuss some of the progress that has been made in using modelling to shape policy, highlighting the challenges facing the field and identifying future research priorities. We do this by first considering how far mathematical models have come in capturing antibiotic resistance evolution and discussing the remaining challenges. Then we evaluate how these models have been successful in guiding decision-making and the questions that remain.

## Main text

### Capturing antibiotic resistance evolution with mathematical models

Before a mathematical model is deployed in decision-making, it must first convince us of its explanatory capabilities. In other words, before a model can be used as a reliable guide for policy, it must be able to recapitulate the empirically-observed prevalence of resistance — typically reported as either the number of cases of resistant infections or the proportion of bacterial isolates exhibiting resistance — at the appropriate local, regional, national or international level. This is not a simple task. Fully capturing these observations ‘from the ground up’ requires understanding: (i) how bacteria acquire resistance, whether by horizontal transfer of resistance genes or de novo mutation [1]; (ii) how these resistant cells proliferate, both within and between hosts; (iii) which forces, including antibiotic exposure, select for the transmission of resistant over non-resistant strains across diverse environments; (iv) how the circulation of resistant strains translates to reported numbers of infections or carriage episodes of resistant strains in different settings, for each “bug-drug” combination; and (v) how diagnostic, sampling, culture and typing methods affect our data on ABR incidence and prevalence.

#### What we know

##### Selection for and against antibiotic resistance

The basis for the dynamics of antibiotic resistance is Darwinian evolution. The presence of an antibiotic selects for a higher frequency of organisms resistant to that antibiotic, because resistance to treatment confers those strains a benefit over susceptible strains [1]. Conversely, many models have assumed that resistance genes impose costs for the bacteria that carry them, resulting in resistant bacteria having lower fitness in the absence of antibiotics [2] — an assumption which is generally, but not universally, borne out by observation [3, 4]. Accordingly, the strength of selection for resistance depends upon the balance between the benefits and costs of resistance. A corollary of assigning a cost to resistance is the ‘time-reversibility’ of evolution — that is, if antibiotic use is removed, resistance is counterselected and should equilibrate to the same frequency as before the introduction of the antibiotic [5]. Further, the between-host transmission of resistant bacterial strains, as opposed to de novo mutation or horizontal acquisition of resistance genes by bacteria, is generally assumed to be an important driver in the maintenance of antibiotic resistance [6]. These principles are naturally articulated within mathematical models that capture the dynamical processes of transmission, colonization, and treatment.

##### Competition (likely) exists between resistant and sensitive strains

While some models of ABR account only for the transmission of resistant strains, there is growing recognition that tracking the dynamics of sensitive strains is important as well [6], especially if these strains are competing for limited resources: a finite niche within an individual person, and a finite number of people to colonise. These competitive dynamics substantially impact resistance evolution in both empirical studies [7–9] and theoretical mathematical models [10–12]. These modelling studies emphasize that competition between resistant and sensitive strains can occur both at the within- and the between-host level, and the relative importance of competition at these two levels can drive resistance evolution in opposing directions [13]. Competition also occurs between commensal and pathogenic bacteria occupying the same niche, with some unculturable competitors that are also affected by antibiotic exposure; this has only recently come to light with the advent of rapid affordable deep sequencing technology and associated analysis [14]. Further theoretical work and empirical investigation will permit a more precise characterisation of the competitive dynamics between resistant and sensitive strains, allowing us to establish ecologically sound principles for modelling competition both within and between hosts.

##### Transmission networks and heterogeneity of exposure to antibiotics

Modelling is beginning to help us understand the geographic networks of ABR transmission [15, 16] in hospitals, communities, agricultural settings, and the environment. Paired with analysis of UK patient movement data, modelling has revealed the importance of locally circulating ABR [17]. Local outbreaks in ABR hotspots such as hospitals and long-term care facilities, which feature high antibiotic use and, often, immunocompromised patients, are generally better documented than broader patterns of community acquisition. Where detailed patient data do exist – often in the intensive care setting – stochastic mathematical models are now being used to assess the extent of transmission attributable directly to patients, healthcare workers or indirectly to the facility’s environment [18]. Combining mathematical and phylodynamic modelling in the advent of cheaper sequence data is likely to present new opportunities to further understand the sources of health care-acquired resistant infections [19]; a better understanding of the role that non-patients and healthcare workers can play in resistance outbreaks may follow. Further, modelling has also been used to suggest that a greater proportion of antibiotic resistant bacteria is acquired in the community than in the hospital setting [20, 21], and hence that antibiotic stewardship efforts should include the community. While the importance of agricultural antibiotic use for human health is debated, modelling results have suggested that curtailing antibiotic growth promotion in livestock will be of less benefit than reducing animal-to-human transmission [22, 23]. Sequence data is likely to further our understanding of transmission from agricultural sources [24, 25]. All told, mathematical modelling is helping us to understand how resistance spreads in specific settings and within specific groups.

#### Challenges remaining

##### Lack of precise understanding of selection pressure

Beyond the empirically well-supported hypothesis that greater antibiotic use by individuals in a population selects for a higher frequency of resistance among bacteria circulating in that population [26, 27], we have not yet convincingly identified the major drivers of the spread of resistance at the population level. One difficulty lies in explaining what maintains coexistence between resistant and non-resistant strains over long periods of time, when simple models predict that, depending upon the average antibiotic consumption rate in a population, either resistant or sensitive strains should competitively exclude the other [6, 28]. A number of recent studies have proposed potentially complementary mechanisms — e.g. balancing selection caused by within-host competition [10, 28], variable selection for resistance over heterogeneous genetic backgrounds [29, 30], or population heterogeneity in treatment rates [28, 31] — which may each be capable of explaining this empirically-observed coexistence [13]. The relative importance of these and other mechanisms will vary depending upon the pathogen and setting, but remains to be identified for any one case.

A further difficulty in characterizing selection pressures for resistance is that a substantial proportion of variation in resistance to specific antibiotics between populations is not explained by variation in the consumption of those antibiotics: identifying interactions between co-selection of resistance determinants [29, 30], bystander selection [31], and other forces selecting for resistance is crucial for a complete understanding of resistance evolution. In principle, model calibration to empirical data could help to choose between alternative mechanisms. There is no shortage of hypotheses for what may contribute to the spread of resistance; what is needed is convincing empirical evidence to identify the most important forces shaping resistance evolution. Finally, on top of these uncertainties, mathematical models overlay a suite of additional and much less understood assumptions — the within-host dynamics of the bacteria within the microbiome, the social mixing patterns of individuals and the existence and strength of coselection. It is precisely the interplay between all these factors that drastically changes what the models actually predict [11, 13].

##### Setting-specific model calibration and data availability

Recent work has begun to calibrate models to empirical data on the relationship between antibiotic consumption and resistance [10, 13], with the aid of databases linking antibiotic use and resistance at a country or state level, such as the European Centre for Disease Prevention and Control’s European Antimicrobial Resistance Surveillance Network (ECDC EARS-Net) [32], the Center for Disease Dynamics, Economics & Policy ResistanceMap [33], and the World Health Organization’s Global Antimicrobial Resistance Surveillance System (GLASS) [34], as well as a host of national surveillance systems. However, these data appear insufficient to distinguish the mechanisms that govern the selection pressure that underpins the dynamics of resistant strains [13]. While, to date, this calibration has only been attempted in the context of a small subset of bacteria-treatment combinations, it is likely that these general limitations will extend to the wider group of pathogens. To distinguish the mechanisms or set of mechanisms generating the resistance dynamics we observe will require investigation of within-host strain diversity, strain epidemiology, and the demography and geography underlying transmission. It will also require consideration of data and properties of a more diverse set of potentially-pathogenic bacteria, as well as commensal and environmental bacteria, than the commonly used example of *Streptococcus pneumoniae* [10, 13, 28, 29, 35].

##### Transmission networks and heterogeneity of exposure to antibiotics

Despite the advances in using mathematical models to disentangle the role of different groups of hosts in the transmission of resistance, elucidating the connections between, and the relative importance of, the heterogeneous environments in which resistance evolves remains a key problem. Both hotspots of ABR acquisition (which could be related to geographical areas/types of food-production systems/healthcare settings) and the most relevant pathways for exposure are unclear. For modelling to inform where to direct interventions, it must span these diverse environments. In doing so, it has the potential to help resolve some of the most contentious debates in ABR policy, such as the relative importance of agricultural, environmental, community and health-care reservoirs as sources of resistant infections.

##### Difficulty in quantifying fitness costs

Open questions remain regarding how to quantify the fitness costs associated with resistance. How large are the costs? Do they manifest as reductions in within-host growth, between-host transmissibility, infectivity, or all three? Mathematical modelling can, in principle, be used to estimate the magnitude of fitness costs associated with resistance directly from epidemiological data [10, 13, 36–39]. However, fitness is a highly location-, time- and strain-specific characteristic [40, 41]; care must be taken not to overgeneralise. As mathematical modelling predictions depend crucially on competitive strain dynamics, which in turn depend on resistance cost, the unknown effect of a combination of synergistic or antagonistic interactions [42] make model predictions highly uncertain. Moreover, although it is implicitly assumed by mathematical models, there is no overwhelming evidence to suggest that costs to resistance genes are unavoidable, whether truly cost-free resistance mutations will eventually arise, or whether back-mutations towards lesser resistance would spread under a reduction of antibiotics.

### Translating mathematical modelling evidence into policy

Mathematical modelling has the potential to test policy interventions in silico, and hence to help us both understand the relevant components in complex systems and assess their relative impact and potential cost-effectiveness both as standalone policies [43–45] and as elements of combination (“bundled”) policies [46]. This approach can then be used to predict the impact of updating the interventions or extending them long term.

#### What we know

##### The usefulness of mathematical models for health policy decision-making

Although a comprehensive overview of the use of mathematical models in health policy [47] cannot be given here, it is clear that infectious disease models currently provide crucial evidence for public health decision making in many areas. A prime example is the use of mathematical models to support vaccination recommendations by National Immunization Technical Advisory Groups (NITAGs), such as the UK’s Joint Committee on Vaccination and Immunisation (JCVI) [48]. In this and other well-established areas of health policy, predictions from mathematical models are translated into health economic terms by expressing health burdens in standardised units, e.g. quality-adjusted life years (QALYs) or disability-adjusted life years (DALYs). This allows the efficiency and affordability of alternative interventions to be assessed and compared in terms of the monetary cost per QALY gained or DALY averted. Although at present this economic framework is not widely used for questions relating to the control of resistant infections, recent estimates of resistance-attributable standardised health burdens [49, 50] are beginning to make this possible [51].

#### Challenges remaining

##### Lack of validated models

As we have discussed above, the widespread use of dynamic modelling is lacking for many current ABR control policies due to the challenges we face in understanding and quantifying ABR transmission [52]. This can mean that we lack a framework for assessing interventions that are rolled out. For example, in the UK, the impact of a recent policy change from broad to narrow spectrum antibiotic usage, with a particular focus on reducing the rate of *C. difficile* infection [53], was not supported with predictive modelling, potentially hampering our ability to optimally assess this intervention in a timely manner. In general, a key function of dynamic models is to predict the time scale on which changes are expected to occur following interventions, and in general this has not been done systematically despite some efforts [13, 54, 55]. As a result, ABR modelling is underdeveloped relative to other areas of infectious disease modelling that support decision-making, such as vaccine policy where model calibration is a key requirement for a model to be fit for purpose [56, 57].

##### Questions of outcomes

In managing the challenge of antibiotic resistance, our goal is not to reduce resistance per se, but to mitigate the health burdens that are caused by resistance. In other words, resistance is only a problem insofar as it leads to worse health outcomes. But how to calculate the attributable health burdens of resistance is an active area of research, and accordingly this remains a barrier to developing informed policy. Rather like climate change, policies must be enacted now to have an impact in the long term [58], but the potential long-term benefits of avoiding resistance must be balanced against the low cost, convenience, and lifesaving potential of antibiotics. Hence, a key area for modelling is the burden of current and future ABR, in terms of morbidity, mortality and economic impact: widely-cited cited projections use have been produced for worldwide ABR burden by 2050 [59], while current and future burden has been estimated in more rigorous frameworks for European countries [50, 60], but better data and methods of attribution are needed to inform parameters such as attributable mortality [49, 61]. Moreover, predictions of future burdens should be tied where possible to a mechanistic understanding of how resistant infection incidence is likely to evolve over time, as described in the prior section.

A complication of quantifying the attributable burden of resistance lies in identifying the counterfactual to a resistant infection: that is, whether calculating the health burden of resistance requires comparing a resistant infection to a susceptible infection, or to no infection at all. This counterfactual would not be the same for all pathogens and settings [62]. The incidence of the syndrome will also vary: for total burden it is the combination of prevalence of resistance with incidence of syndrome that matters, and these in turn may be affected by rates of antibiotic use and/or prevalence of resistance [63]. Reducing this complexity down to an index that can be easily communicated can give insight into how resistance levels are changing in time and space [64]. These estimates are important not only for policy makers, but also for properly incentivizing the development of new antibiotics.

##### The case of antibiotic stewardship

A key intervention is antibiotic stewardship: preserving the efficacy of antibiotics by limiting their unnecessary use, optimising dosages and durations of treatment, and using drugs or combinations of drugs that limit selection for resistance. A major impediment to effective stewardship is that we do not know exactly what features of antibiotic use — drug, dosage, length versus frequency of treatment episodes — are most important for promoting resistance, and yet these factors may have a significant impact upon resistance evolution (e.g. [35, 65]) and could help to explain the variation in resistance between settings that is not explained by the volume of antibiotic consumption alone. This may be due to the complexity of determining how to measure resistance and antibiotic consumption, how to weigh up the importance of antibiotic use across different populations (e.g. humans versus livestock), and what constitutes “appropriate” treatment [66].

In some settings, policies reducing antibiotic use—either overall or within specifically targeted classes— have been associated with reductions in resistance [67–71]; however, these conclusions have not been universal (e.g. [72, 73]). Results vary due to key unknowns: notably, whether reduced antibiotic use will always reduce resistance, at what rate increased use will increase resistance, whether a given population is at equilibrium resistance prevalence and how fast these equilibria are reached. With this level of uncertainty, mechanistic models are often unable to robustly capture the dynamics and instead statistical trend prediction or machine learning has been employed. For example, in analysing trends for 72 pathogen-antibiotic combinations across the United States, statistical modelling has recently suggested that broadly-distributed, low-intensity use was more strongly associated with resistance levels than repeated use of antibiotics [74]. Since repeated use might represent the ‘low-hanging fruit’ of antibiotic stewardship efforts, this finding highlights a potential policy challenge.

One area where discussion of ABR policy has been most led by mathematical models is the long-standing debate over whether rotating antibiotics (that is cycling the use of a single antibiotic class within a single population) or using different combinations (mixing antibiotic classes within one population or combining antibiotics classes within individual patients) better prevents resistance acquisition (see [6] for a wider discussion). Diverse predictions provide insight into underlying process, but prevent universal conclusions from being drawn and modelling may be best viewed as complementary to clinical trials [75]. This highlights how far we have to go to understand the selection and transmission of resistance under antibiotic treatment.

##### The case of vaccination

Vaccination has been proposed as a means of mitigating the burden of resistant infections [59]. Bacterial vaccines can be used to prevent infections that may otherwise require treatment with antibiotics, while viral vaccines can prevent diseases such as influenza which are often treated inappropriately with antibiotics. Mathematical frameworks have been developed for modelling the broader reduction in prevalence of infection due to vaccines [59, 76], as well as for estimating the impact of viral vaccines on antibiotic use and resistance [51]. However, the long-term impact of bacterial vaccination on the evolution of antibiotic resistance is complex (reviewed in [45]), and uncertainties over what drives resistance evolution lead to varying predictions concerning whether vaccination inhibits or promotes the long-term evolution of antibiotic resistance, where the nature of competition between resistant and sensitive strains has been identified as crucial for determining the impact of bacterial vaccination on resistance [13, 77].

##### The case of diagnostic tests

The promise of rapid diagnostic tests — or substitutes such as machine-learning-guided clinical histories [78] – is the potential to alleviate some of the uncertainties surrounding which antibiotics should be prescribed for a suspected bacterial infection. Nonetheless, the evidence of clinical impact on antibiotic use is sparse [78–80] and few studies have investigated the impact on antibiotic use or resistance [52]. In this situation, modellers must work closely with microbiologists and clinicians to develop tools that correctly capture what is being empirically measured as well as guiding surveillance system design; only then can models precisely determine the relative impact of interventions. It will be important to distinguish the short-term benefits of optimizing treatment [78] from the longer-term effects of more appropriate treatment on the evolution of resistance [81].

##### The case of clinical trials

A significant barrier to determine competing risks of policy interventions is the lack of standardisation of resistance outcomes in current clinical trials. There is a limited number of strategic trials comparing alternative antibiotic regimens, but the majority either do not measure ABR outcomes at all, or compare different types of clinical samples, taken at different times, with widely varying phenotypic and genotypic methods. This makes comparison between studies very difficult and prevents the assessment of optimal outcomes from an “ABR perspective”. There is an urgent need to provide some harmonisation and guidance on assessment of resistance outcomes - including some early form of standardisation of units of resistance at an individual and population level [64]. Major policy interventions under consideration, for example, mass drug treatment with azithromycin [82], will include formal drug toxicity and clinical cost benefit assessments, but cannot currently include any formal assessment of adverse effects on drug resistance in the population as there is no standard methodology to use. This has the effect of downgrading potential ABR adverse outcomes, with policy decisions driven by cost/toxicity factors that can be formally measured. This inevitably limits the modelling support that can be done to aid intervention design for ABR control.

##### Prioritizing resources

Determining where to target policies — for example, towards the agricultural community or at interventions such as improved sanitation — is hampered by a lack of quantification of the source and drivers of ABR [83]. Building modelling into established protocols for decision making, such as is done for vaccines in the UK [48] and formally assessing interventions as they are rolled out would improve decision making. In particular, models that determine the differential impact of interventions by geographical setting could be used to inform the development of national action plans [84].

##### Surveillance of trends

Fundamentally, modelling for policy requires some assessment of trend: to improve modelling requires better granular surveillance data on trends in ABR in different environments (e.g. [85]) as well as a better understanding of the relationships driving these trends. Reduction in ABR must also be consistent with policy aims—such as reducing overall infection prevalence or mortality—which may require increasing the use of antibiotics. Modellers must be sensitive not only to ABR dynamics, but the context within which a given policy sits.

## Conclusions

Mathematical models are needed to make good decisions about how to manage ABR, because they make understanding the complexities of resistance evolution more manageable. Therefore, the mechanistic framework of mathematical models provides a valuable opportunity to both quantify ABR transmission and understand how to optimise usage of antibiotics and other interventions. Mechanistic models implicitly capture aspects of antibiotic resistance that we find more intuitive, such as the selection of antibiotic resistance in the presence of antibiotics and the existence of fitness costs of resistance. Accordingly, mathematical models can also help us to formulate novel ways of managing resistance.

However, the current state of mathematical modelling of ABR has both conceptual and empirical gaps, which urgently need to be filled given the importance of having good models. Model results tell us that details matter: the strength of selection, the type and strength of fitness costs and the extent of competition between resistant and sensitive strains all change the dynamics of resistance evolution. However, without being able to routinely inform and calibrate these models with comprehensive epidemiologic data, we currently lack confidence in model predictions, most notably at the larger regional and national scale. The potential drivers of resistance evolution that have been supported by or identified using mathematical models are numerous. Empirically testing these hypotheses would allow us to identify the mechanisms that really matter for informing policy.

Hence, whilst modelling has already been useful for developing policy in other areas of infectious disease control and, as such, there exist frameworks for integrating model predictions into an economic evaluation, there is much more to be done before mathematical modelling can robustly underpin ABR control policy. With this in mind, we propose three key goals (Table 1) that, if achieved, will help inform research across the ABR control strategy portfolio.

**Table 1:** Priority areas for ABR mathematical modelling to inform policy

(1) Explaining population level resistance trends: testing and combining current model structures with diverse multi-level datasets. While there exists a suite of plausible mechanisms that may drive trends in resistance evolution, we currently lack the empirical data to evaluate the relative importance of these mechanisms. To resolve this difficulty, we will have to collect these data and systematically calibrate the suite of models to these data. Doing so will allow us to not only distinguish the underlying mechanism(s), but also to quantify other key parameters such as the strength of selection and competition for a particular bacteria and drug.	
(2) Disentangling transmission routes: fitting models to data to generate a standardised modelling framework that shows the pathways of ABR will help to improve intervention targeting as well as to predict future burden.	
(3) Translating model predictions to economic outcomes: evaluating the cost-effectiveness of competing ABR control strategies. Although the framework for integrating mathematical model predictions into economic frameworks exists in principle, more work is needed to develop methods specific to antibiotic resistance, such as calculating the short- and long-term costs of antibiotic resistance across priority pathogens and correctly identifying counterfactual scenarios that are contingent on the epidemiology of the pathogen and its setting. Adopting a standardized approach for evaluating the efficiency and optimality of strategies would be invaluable across hospital settings, where arguably the need is greatest.	

## Data Availability

Not applicable.

## Abbreviations

ABR: Antibiotic resistance
DALYs: Disability-adjusted life years
ECDC EARS-Net: European Centre for Disease Prevention and Control’s European Antimicrobial Resistance Surveillance Network
GLASS: World Health Organization’s Global Antimicrobial Resistance Surveillance System
JCVI: UK Joint Committee on Vaccination and Immunisation
NITAGs: National Immunization Technical Advisory Groups
QALYs: Quality-adjusted life years
